# Prenatal, Delivery and Postpartum Care Experiences among Black Women in Mississippi during COVID-19 Pandemic 2020–2021

**DOI:** 10.3390/women3020022

**Published:** 2023-05-24

**Authors:** Praise Ebimaye Tangbe, Mary Shaw-Ridley, Gerri Cannon-Smith, Sheila McKinney, Nelson Atehortua, Russell Bennett

**Affiliations:** 1 College of Health Sciences, Jackson State University, Jackson, MS 39213, USA; 2 Mississippi State Department of Health Consultant, Jackson, MS 39216, USA

**Keywords:** pregnancy, experiences, concerns, COVID-19 pregnancy script, black women, maternal mental health, attitudes

## Abstract

The COVID-19 pandemic has presented challenges for countries to maintain high-quality, essential maternal health services, altering pregnancy experiences for women. This qualitative study aims to explore the impact of COVID-19 mitigation strategies on self-reported prenatal, delivery, and postpartum care experiences among Black women in Mississippi. Postpartum Black women who gave birth between March 2020 and March 2021 were recruited from a Federally Qualified Health Clinic that serves three Mississippi counties. Using a semi-structured interview guide, 10 postpartum women were interviewed, and their responses were analyzed utilizing the thematic content analysis approach. Major themes identified were stress related to COVID-19, disruption of social life/support, disruption of expected healthcare services, uncertainty and fear about coronavirus, COVID-19 mitigation strategies, and associated poor maternal health outcome. COVID-19 mitigation strategies exacerbated normal maternity-related stress. Postpartum women reported increased anxiety, fear, frustration, emotional stress, and lack of social support resulting in what was described as depression and feelings of loneliness. The results of this qualitative study of 10 Black women who gave birth during COVID-19 suggest the importance of stress-informed care.

## 1. Introduction

Maternal morbidity and mortality are prominent global public health challenges, and the coronavirus pandemic has increased difficulties in delivering optimal prenatal, delivery, and postpartum services [1]. COVID-19 exacerbated maternal health disparities and undermined maternal health [2]. COVID-19 mitigation strategies restricted prenatal care visits, triggering feelings of isolation that may have contributed to higher rates of postpartum depression. Pregnancies of Black women were found to be disproportionately affected [3,4]. Vaccine hesitancy led to relatively low rates of COVID-19 vaccine uptake in this population [5], resulting in increased rates of maternal morbidity and mortality [6]. One study pointed out that Black pregnant women in the United Kingdom showed significantly higher rates of maternal mortality than White mothers [7]. Another similar study showed that pregnant Black women in the U.S. who opted for telehealth prenatal care experienced challenges [4]. They did not receive quality care that included essential tests or necessary in-person visits [4,8], which worsened already existing healthcare inequities [9]. As of April 2021, approximately 86,877 pregnant women in the U.S. were infected with coronavirus and 97 died [5]. Due to the physiological changes in cardiopulmonary systems and immune systems during pregnancy, the severity of COVID-19-related illness increases [10]. Previous coronavirus outbreaks had already suggested that pregnant women and their fetuses are particularly susceptible [11]. Reported complications are stillbirth, preterm birth, and maternal mortality [12]. In addition, there is an increased risk for cesarean section, postpartum hemorrhage, preterm birth, and hypertensive crises [13]. Mitigation strategies such as social distancing, self-isolation, quarantining, and face masks [3] interfere with prenatal care, labor and delivery, and postpartum care [10,14]. The pregnancy experience during COVID-19 may have long-term implications for the health and well-being of mothers and their children [14]. In Mississippi, where maternal morbidity and mortality rates are among the highest in the U.S., there are no known studies on the COVID-19 lived experiences of Black pregnant women despite the burden of maternal morbidity and mortality in this population [15]. Therefore, this qualitative study aims to (a) explore Black women’s experiences with prenatal, delivery, and postpartum care during the COVID-19 pandemic, and (b) the effects of mitigation strategies. It hopes to generate themes that will be able to guide maternal stress-informed care during crises such as those experienced during COVID-19.

## 2. Methods

### 2.1. Protection of Human Participants

This study received ethics approval from the Jackson State University Institutional Review Board. Additionally, this study required written informed consent and participants’ were informed of their right to decline participation at any time during the interview.

### 2.2. Maternal Healthcare Framework

The Maternal Healthcare Framework (MHCF) was adapted [16] to guide the study design. The model provides the framework for exploring the COVID-19 impact on prenatal, delivery, and postpartum care among Black postpartum women.

- Prenatal care experiences: The pandemic led to limited access to healthcare facilities, which caused limitations and restrictions in prenatal visits [17]. Black postpartum women express their lived experiences with prenatal care during the coronavirus pandemic.
- Delivery care: COVID-19 impacted hospitals by restricting visitation to hospitalized patients to support mandated social distancing. According to [18], most maternity wards allowed solely the woman’s partner in the delivery room. Therefore, a critical element of the MHCF is to have mothers reflect on and describe their childbirth (delivery) experience during the COVID-19 pandemic.
- Postpartum care support: There is a realization that the COVID-19 mitigation strategies were important to curb the spread of coronavirus, but the lack of social support was a non-intended outcome. Most hospitals took precautions by prohibiting visits during postpartum hospital stay [18]. Moreover, the implemented stay-in-place measures prevented visits by family members and friends and limited face-to-face care management by caregivers. These restrictions placed mothers with newborns in an emotionally harmful, psychologically vulnerable space that increased risk of postpartum depression. Therefore, postpartum mothers expressed their lived experiences with the coronavirus pandemic and postpartum care support.

**Figure 1.**
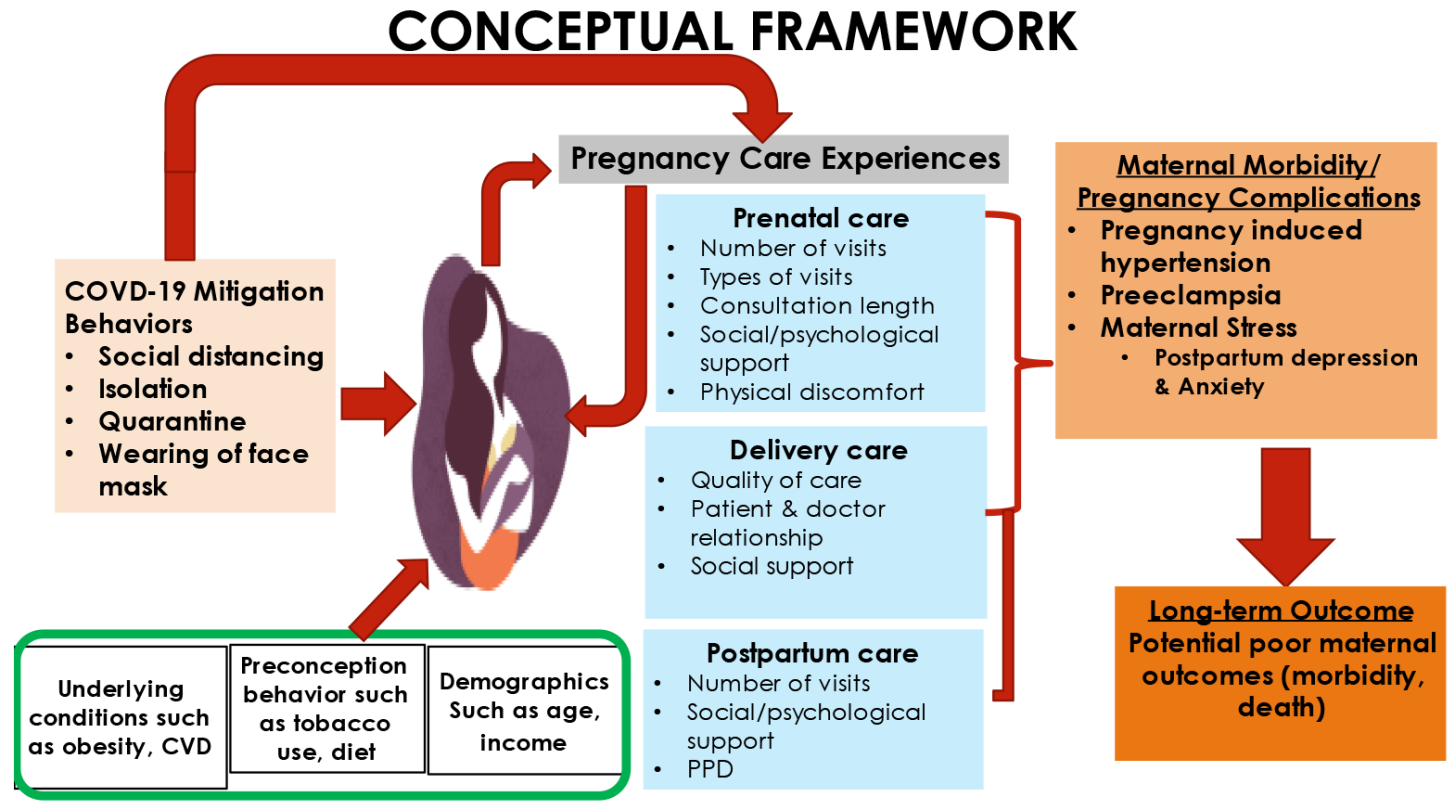
Maternal Healthcare Framework (MHCF). Source: The Maternal Healthcare Framework © 2022 Praise Ebimaye Tangbe was adapted from “Chronic Disease Management: What will it take to improve care for chronic illness?” by Wagner E. H., 1998, *Effective clinical practices, vol 1* [16].

The MHCF model situates prenatal, delivery, and postpartum care experiences of Black women within a culture (both societal and hospital) that has COVID-19 mitigation strategies/behaviors in place (see Figure 1).

### 2.3. Study Design

We conducted a cross-sectional qualitative study using a narrative approach to explore participants’ experiences with prenatal, delivery, and postpartum care services during the COVID-19 pandemic (March 2020–March 2021).

### 2.4. Population and Sample

Using convenience sampling, participants were (a) women between ages 18 and 45 years; (b) postpartum women who gave birth between March 2020 and March 2021; (c) people self-identifying as Black or African American in Mississippi; (d) postpartum women who spoke and understand English. Participants were separated into three age groups (18 to 24, 25 to 34, and 35 to 45) because, in the U.S., the prevalence of preterm birth is higher among women < 20 years of age (young maternal age) and women between the ages of 35 and 45 (advanced maternal age) compared to women in their mid-twenties to early thirties. Moreover, in this qualitative study, the saturation points guided determination of the final sample size [19,20]. Therefore, data saturation was reached with ten participants’ responses.

### 2.5. Instrumentation

The researcher developed pregnancy experience instrument is based on the WRISK survey [21]. In a two-round process, the researchers developed an interview guide that was validated for content validity, face validity, and readability by a panel of three qualitative research experts. In addition, the instrument was pilot-tested with three Black postpartum women (from the same clinic where participants were recruited) to evaluate study procedures and develop appropriate probes for study interviews. The instrument consists of two sections: demographic profile and a semi-structured interview guide with three parts (prenatal care, delivery care, and postnatal care support).

#### Demographic Profile.

The first section consists of eight items, representing the participant demographics: age, marital status, education level, employment status, income, insurance status, gestational age, and number of pregnancies.

#### Pregnancy Experiences.

The second section of the instrument is organized into three parts consisting of six items that assess prenatal care during COVID-19, eight items that assess delivery care during COVID-19, and seven items that assess postpartum care support during COVID-19.

### 2.6. Recruitment Strategy

Recruitment occurred between 1 December and 15 December 2021 (fifth wave of COVID-19 during the omicron variant surge). We recruited women receiving postpartum care support from a Jackson Metropolitan area Family Health Center (FQHC). The site is a Federally Qualified Health Center that serves about 2900 low-income rural and urban pregnant and postpartum women in three counties (Madison, Humphreys, and Yazoo). During the first wave of the pandemic, the Family Health Center strictly enforced the CDC COVID-19 mitigation guidelines [3] in care facilities. For example, prenatal and postpartum visitations were moved from in-person to virtual; the Women Infant Children (WIC) program weekly meetings were temporarily canceled; and wearing of face masks, regular hand washing/sanitation, and staying 6 feet apart were made compulsory in the facility. Eventually, the researcher and the clinic physicians disseminated recruitment cards during reinstated WIC weekly program meetings and doctor’s appointments. Interested postpartum women contacted the primary investigator by phone. Postpartum women were screened via phone using a three-item screening eligibility tool. Eligible women were enrolled in the study and scheduled for an interview (face-to-face on site or virtual) one week later.

### 2.7. Data Collection Procedures

Interviews were conducted in person and virtually for five weeks, from 1 February 2022 to 7 March 2022. A team of two trained interviewers including the researcher were available to conduct the interviews. Each participant consented prior to the beginning of the interview. The researcher discussed the details of the study with the participants before proceeding with the interview. For interviews via Zoom, informed consent was emailed to each enrolled participant one week before the interview date. Moreover, participants were required to consent to audio recordings. One interviewer asked the interview questions, and the other took notes and observed participants’ responses during the conversation, such as tone of voice, non-verbal body languages, and important notes about the interview. Each interviews lasted approximately 30 to 45 min. Two interviews were conducted in person, and eight interviews were conducted virtually. Each participant received a 20-dollar gift card incentive upon completion of the interview.

### 2.8. Data Analysis

#### 2.8.1. Quantitative

The Statistical Package for Social Sciences (SPSS) version 27 was used to analyze the demographic data. Descriptive statistics were used to report demographic information.

#### 2.8.2. Qualitative

Data were transcribed using the online Rev transcription services. The transcripts were then entered into an Excel spreadsheet. Next, thematic content analysis was employed to analyze the presence, meanings, and relationships across the dataset that provided answers to the research questions. We identified these patterns through (a) data collection familiarization, (b) coding data, (c) applying templates of codes, (d) connecting codes and identifying themes, and (e) validating themes.

##### First stage (data collection and familiarization).

We reviewed transcripts to become familiar with the dataset. This process helped identify meaningful units characterizing participants’ prenatal, delivery, and postpartum experiences during COVID-19.

##### Second stage (coding the data).

Members of the data-analysis team independently reviewed the text and developed codes. We then met several times to determine preliminary codes by reviewing highlighted meaningful units. Once the frequently used words and phrases were identified and the phenomenon was captured, the codes were categorized based on their differences and similarities in addressing participants’ responses.

##### Third stage (applying template of codes).

The Codebook was developed utilizing Microsoft Spreadsheet. Preliminary codes were inserted in the Excel spreadsheet. Each interview question included a set of codes with supporting participants’ responses on prenatal, delivery, and postpartum care experiences.

##### Fourth stage (connecting codes and identifying themes).

The codes were categorized into themes and patterns in the data. The themes permitted comparison of pregnancy experiences (prenatal, delivery, and postpartum) during COVID-19.

##### Fifth stage (corroborating and validating themes).

This stage included the process of clustering the themes that were identified from the coded text. The aim was to identify the essence of each theme and to establish that they were representative of the preliminary assigned code. We conducted several iterations of reviewing text, codes, and themes, then agreed on succinct and easily understandable words/phrases for each theme before moving to the interpretive phase of the analysis.

## 3. Results

### 3.1. Demographic Characteristics of Participants

The results are discussed in two sections. First, the demographic characteristics of the participants are presented. Secondly, major findings from the thematic content analysis are presented in a qualitative style format. Table 1 shows the demographic profile of the participants.

### 3.2. Participants’ Interview Responses

The five major themes that emerged about prenatal care, delivery care, and postpartum support are the following: stress related to COVID-19, disruption of social life/support, disruption of expected healthcare services, uncertainty and fear about coronavirus, COVID-19 mitigation strategies-associated poor maternal health outcome.

Table 2 shows the five derived themes with supporting quotes. The study results show how a sample of Black postpartum women characterized the prenatal, delivery care, and postpartum support experiences during the COVID-19 pandemic.

#### 3.2.1. Theme 1: Stress Related to COVID-19

Postpartum women reported feelings of stress during the COVID-19 pandemic with experiences in prenatal, delivery, and postpartum care. Participants reported they had been stressed and frustrated to have to take care of their newborn by themselves. The fact that hospitals limited family members and spouses from attending prenatal, delivery, and postpartum visits was distressing for women and affected their emotional state of mind. Participants also indicated physical stress and discomfort from wearing face masks. The following selected quotes support the theme that emerged.

“Somehow stressed, emotional stress of thinking of the uncertainty of what the virus would do to the child.”

“It was tough, even though I enjoyed lockdown but just being confined to the house with a newborn Umm and still having to like work from home, it was tough. There was moment of frustrations.”

“The COVID-19 restrictions when going to the hospital, it was a little bit stressful.”

“I was kind of nervous about everything,” “it was a little bit stressful.”

“It was a bit stressful you know with the sleepless nights and then trying to get better take care of the baby and do everything by yourself without people around, It was a little you know exhausting.”

“I think it was a little more stressful because I had three kids to take care of, and the mask I was wearing also made it stressful. Very uncomfortable and stressful because at every point in time, we also have to mask up, you’re sleeping and somebody comes into your room, you have to wake up and mask up.”

“Umm stressful, anxious and frustrated and I was moody, very moody but not a bad temper but kind of snappy.”

“Frustrated and stressed and COVID was around and I had to deliver during COVID and I had to dislike wearing face mask.”

“Wearing masks are stressful and uncomfortable and when you’re pregnant, especially when you start getting to the second and third trimester it’s very, when walking you know, sometimes you feel breathless.”

#### 3.2.2. Theme 2: Disruption of Social Life/Support

Participants indicated that they were not being sufficiently supported during the COVID-19 pandemic due to the mitigation strategies. They complained of lack of support from family and friends. The fact that hospitals restricted family members and spouses from attending prenatal, delivery, and postpartum visits was distressing for the participants and affected their emotional state of mind.

“They had to limit people coming into the room to just one person to know like I know before the pandemic, you will have your maybe your parents your spouse, but you just have to have only one person present. It was out, personally I wasn’t happy with it because you Know, I like that experience I wanted family and my spouse to be present but it just had to it was one or the other.”

“It was bad to not have, like in during pre-COVID you could have as many people in the room as you want to, but during the COVID I am with one person and you know not having your mom or sister around when you deliver was kind of tough.”

“I feel very much separated, being away from family and friends.”

“I didn’t like the experience that much people where not allow to be with me during delivery, I actually wanted my step mom to be with me because this is my first child.”

“I felt a little I guess alone because people didn’t visit that much and I couldn’t visit as well.”

“I could only choose one person to be with me, but when I delivered him, it was his dad and my mom. It was just weird because I just remember everything, even my friends who have kids you know before COVID, everybody was just in the hospital, you know celebrating the baby and stuff, mine was just so different because you know you can’t invite people because of COVID.”

“Umm not being able to go anywhere and not being able to have anyone come around, and whenever somebody kind of bring something they would leave it on the doorstep, so I feel like I have to play by yourself and like people had to avoid me at all costs, so I felt very isolated.”

“It was a little sad, because due to social distancing I couldn’t go visit people and that made me sad.”

“At home, I felt a little I guess alone because people didn’t visit that much.”

“I feel very much separated, being away from family and friends.”

#### 3.2.3. Theme 3: Disruption of Expected Healthcare Services

Postpartum women mentioned some disruptions in receiving healthcare services in the beginnings of the pandemic such as cancellation of pregnancy-related appointments, the wait times to see a doctor and for visits in the hospital, delay in labor induction, and hospitalization stay after delivery was shortened.

“I like the way they handle their social distancing procedures but the waiting period was a long one.”

“What I can say is that they won’t allow you to have your baby until like 39 weeks. They stopped inducing; before COVID-19 they can induce people Okay, but now because of COVID-19, they do not induce people; they want you to get ready, maybe baby almost you know. they will want you to come to the hospital for delivery when you are sure is your due day. This was because they don’t want too much people to be in the hospital at the same time.”

“It was a long wait, the doctor I was seeing had a lot of patients, a patient was about to have a baby so he had to go there, so I either wait or reschedule.”

“They made it in way that once you put to birth within 24 h you are to leave for another person to come in. It was a really tough one.”

“They limit their appointment time or the number of people who are present during each appointment. Just to reduce like exposure to other people may have COVID.”

“I guess unavailability of scheduling by not been able to get a visit within the week I needed to come because of the COVID it may have been delayed, so I will be coming two weeks after.”

“So, I was really concern I don’t want to get COVID while pregnant. My prenatal appointment was cancelled by the Doctor and rescheduled for another date.”

“It was just really the delivery part and about like being sent home too early, because of COVID guidelines. I came to the hospital on 25th night, gave birth to my child on the 26th and I was back home on the 27th. I just feel like there wasn’t enough time to monitor and make sure I am OK before sending me home.”

“I feel badly, because is a new protocols and people don’t stay in the hospital as long as they used to before COVID. Is like you had a baby, are you feeling good? Okay time for you to go home, I feel like I should have stayed longer, but I didn’t. I feel like I was failed honestly because first of all, they didn’t tell me that my blood pressure was high, or what signs to look out for if I need to come back or call, and you know something like that they didn’t educate me on it.”

“I will give it a 7 over 10 because I think they could do better with follow-up. It was just two postpartum follow-ups.”

#### 3.2.4. Theme 4: Uncertainty and Fear about Coronavirus

Most of the participants were worried and scared about the risk of getting infected by COVID-19 and how pregnant women are more vulnerable to COVID-19 compared to non-pregnant women. Some of the participants’ responses were:

“I was nervous. I think spend the first three to four months within, unless it was a doctor appointment, because I was afraid of the COVID.”

“Umm it was scary, there was lot of uncertainties if I want to deliver in the hospital, so there was like my anxiety level high at sometimes.”

“I am always scared of getting sick, so it won’t affect my baby, I try to stay six feet away from people.”

“Not wanting to get COVID-19, there was like a barrier to not want to go out or not want to go see the doctor because other people may be sick.”

“Hmm … It was it was scary because you know. It was something new and unfounded and I especially had concerns because, you know being pregnant, even though there was a vaccine, I chose not to take the vaccine, because I did, there was no research on you know the impact of it on pregnant women or the baby.”

“There were some scary parts like getting COVID and all that, which made me to miss some of my doctor’s appointment you know.”

“Somehow stressed, emotional stress of thinking of the uncertainty of what the virus would do to the child.”

#### 3.2.5. Theme 5: COVID-19 Mitigation Strategies-Associated Poor Maternal Health Outcome

Participants explained the challenges they faced post-delivery due to the social distance measure enforced to curb the spread of COVID-19. Participants reported significant reduction in postpartum follow-ups and lack of social support from family and friends, which resulted in poor maternal health outcomes such as postpartum depression and preeclampsia.

“Um I think that’s where my postpartum depression came from, because you know, for a while you can’t leave the house and I actually have a baby, and it was just weird because it felt like this is how is going to be every single day and I barely go to anywhere anyway, because I was still terrified of COVID.”

“I was breastfeeding also in pain after given birth I have forgotten what is called, it had to enter my body for 24 h, so I couldn’t get up for 24 h, it was miserable. I did experience postpartum depression.”

“I had bad postpartum depression, so I was put on some different meds to help with that. I was on Zoloft for a little short period. I was just frustrated at that time because I had to take care of my newborn and my other two children myself.”

“My care was a bit quick they somehow rushed me out of the hospital because of COVID. I was admitted again to the hospital, and I was told that I developed preeclampsia, so I had to stay in hospital for three days, because my blood pressure was extremely high.”

In addition, participants recommended proactiveness from care providers during crises, doctors should provide sufficient sources of information, increasing the number of healthcare providers/facilities to accommodate pregnant women in times of crisis, quality health insurance by improving welfare packages during crises, providing mental health support services for pregnant women during crises, improving on doctor–patient relationships during crises, and care providers should have action plans during crises to prevent delays in services among pregnant women. Some of the participants’ responses were:

“We should be more proactive with public health; a lot of misinformation that is available so. clinicians should be in the habit of you know. Advising their patients on the truth about COVID. Because there’s a lot of people who still believe that it’s a myth or it’s real.”

“They need to increase the number of hospitals and also the number of healthcare providers. They should improve on the welfare package for insurance.”

“They dig deeper to connect women to the support services that they may be in the future so that whenever you do feel lonely and isolated or frustrated you do not only have people in place to talk to, but you also have access to tools and resources that you may need to help get you through those tough times.”

“Doctors should try and get closer to their patients, try to know them one on one and also try to impact positively in their life.”

## 4. Discussion

In this exploratory study, the researcher examined pregnancy experiences (prenatal care, delivery care, and postpartum support care) among Black women who were pregnant, delivered, and/or were postpartum during the first year of COVID-19. Interviews were conducted until data saturation occurred (the extent to which no new code could be extracted) from the data. In this study, data saturation was reached with 10 postpartum women. The study findings provide significant insight into Black postpartum women’s lived experiences during the COVID-19 pandemic. Postpartum women (who received prenatal, delivery, or postpartum care during the period March 2020–March 2021) described how the COVID-19 pandemic unexpectedly changed the standards of care processes for prenatal, delivery, and postpartum care support. Emotional/physical stress, disruption of social life/support, disruption of expected healthcare services, uncertainty and fear about coronavirus, COVID-19 mitigation strategies associated with poor maternal health outcomes, feelings of depression, and loneliness were common among postpartum women during the COVID-19 pandemic. Most of the women in this study expressed that they could not keep up with their old social lifestyle because the pandemic disrupted their social lives and support. Socially isolating themselves at home and not having visitors during delivery and the postpartum period led to feelings of loneliness. These findings are like results reported by [22-25]. The current study corroborates the emerging evidence that the COVID-19 mitigation strategies exacerbated maternity stress and levels of depression among pregnant and postpartum women, including Black women who are disproportionately impacted by maternity stressors.

Furthermore, postpartum women reported limited social interaction with other pregnant women during prenatal care visits and how social distancing made them feel isolated, which resulted in loneliness. Postpartum women felt that they were not sufficiently supported during prenatal care because they were not allowed to have their spouses or family members attend prenatal visits. Moreover, in another study women reported a sense of loneliness and lack of support [26]. The findings are similar to what women reported in this study.

The study participants reported feelings of stress or they felt stress related to the COVID-19 pandemic. The study revealed how postpartum women were constantly stressed and nervous about everything; participants reported that they were emotionally stressed and had high anxiety. Emotional stress from participants resulted from mood swings and frustration that resulted from a sense of helplessness. Anxiety, a kind of psychological stress that triggers a physiological state and causes a decrease in immunity and increases the production of stress hormones [27]. Study findings also show that postpartum women reported physical stress and discomfort; difficulty in breathing, were frustrated, inconvenienced, and were uncomfortable wearing masks during pregnancy—perhaps causing anxiety symptoms. The participant responses were similar to what was reported by other groups in the general public (non-pregnant people).

The findings of this study show that the COVID-19 mitigation strategies that were enforced to curb the spread of the virus, led to disruption of social life/support for the postpartum women. Overall, participants felt unsupported, lonely, and abandoned due to a perceived lack of family/friends’ support. The participants in this study reported their experiences during delivery care. The most common response about the lived experiences with delivery care was about the limited number of persons such as spouses, friends, and family members in the delivery or patient room during the COVID-19 pandemic. Postpartum women explained that they were concerned and worried about spouses not being allowed into the labor room. This finding is like the results of [24]. Participants further explained that they felt lonely and isolated at some point in the delivery room due to the limited number of persons for visitations.

During the postpartum period, women explained that social isolation mitigation strategies resulted in either a complete lack of support from community and family or a significant reduction in postpartum and social support, findings that are very similar to results reported by [28,29]. The literature provides evidence that postpartum women with low social support had higher chances of developing postpartum depression than those with high social support [30,31]. Social support from family members, friends, or significant others is prominent in reducing stress and preventing depression during the prenatal and postpartum periods [32,33].

During the maternal care period, Black postpartum women expressed their experiences with the delivery of healthcare services during the COVID-19 pandemic. Postpartum women in this study reported long wait times at the doctor’s office, limited appointments to see doctors, and cancellations of appointments. These findings are similar to the findings of [25,26], which reported that women experienced a reduction and postponement of prenatal care visits. Additionally, hospitalization stays after delivery were shortened; postpartum women stated that they were sent home 24 h after delivery. The response from postpartum women in this study is similar to the findings from [34], which stated that short birth hospitalization length of stay increased from 28.5% to 43.0% for all births (less than two midnights for vaginal deliveries and less than three midnights for cesarean deliveries) [31]. These expected healthcare changes were reported in a previous study, which included cancellation of appointments and restriction on family/friend support during visits to hospitals.

At the beginning of the COVID-19 pandemic, postpartum women were scared about the virus because of misinformation and how pregnant women were more at risk to be infected by COVID-19 compared to non-pregnant women. Postpartum women were concerned about getting infected with coronavirus and afraid about having a healthy delivery without infecting the fetus. These findings are similar to an earlier study, which reported that pregnant women were scared and worried about delivering in the hospital because of the spread of the virus [34]. These experiences affected pregnant women’s choices and created uncertainty and fear about their pregnancy and childbirth care.

Thus, pregnant women who experience high stress are prone to hypertension and other pregnancy complications [5,28]. A previous study reported similar results, showing the effects of the COVID-19 pandemic caused depression, which led to increased anxiety levels among pregnant women [29]. Several participants explained the challenges they have faced post-delivery, such as a limited number of postpartum follow-ups and a lack of support from family members, which resulted in postpartum depression and preeclampsia among some participants (the participant felt she should have been in the hospital for more than 24 h to observe her health because she was diagnosed with high blood pressure. Instead, she was discharged without further observation due to the hospital-imposed COVID-19 restrictions; her blood pressure was elevated, which may have caused her to develop preeclampsia). Other studies reported similar findings, stating that the COVID-19 pandemic caused limited social support and home visitations for women; moreover, pregnant women reported more depressive symptoms in the postpartum period [23,26]. The pandemic exacerbated what were already poor maternal outcomes in the United States.

### 4.1. Strengths

The study is the first to explore the pregnancy experiences of Black women in Mississippi during a selected period of the COVID-19 crisis. The findings characterized the lived experiences of a convenience sample of Black women who received prenatal, delivery, and postpartum care at a Mississippi Family Health Clinic (FHQC) during March 2020–March 2021, the first year of the pandemic. Results showed that the postpartum women described high levels of emotional stress, anxiety, feelings of isolation, and being cut off from family support and a social life that they deemed essential to their well-being. The participants reported similar experiences regardless of whether they were pregnant for the first time, or they had had previous pregnancies. Although the COVID-19 pandemic has impacted everyone, pregnancy has always been a unique life-altering personal, family, and healthcare experience for women. It is unclear what will be the pandemic’s long-term on Black women’s health and future pregnancies for women who experienced prenatal, delivery, and postpartum care during the pandemic. What we do know is women had elevated levels of stress, anxiety, depression, and feelings of isolation that could potentially impact future pregnancies and require a more formal process of stress-informed care by women’s healthcare providers, inclusive of obstetricians, gynecologists, pediatricians, and other primary care physicians.

### 4.2. Limitations

Despite the new findings and emerging evidence from other research, this study has some limitations. These include a small sample size that limits generalizability to all postpartum women in Mississippi and beyond. Secondly, this qualitative study only included English-speaking participants. The sample was limited to one Family Health Clinic (FQHC) in Mississippi that serves low-income women. The findings may not be generalizable to women receiving care services through all healthcare facilities in Mississippi. Moreover, the use of convenience sampling may introduce a high level of bias and inability to generalize findings. The researchers also observed that the participants turned off their video cameras during virtual interviews. It took much work for the interviewer and observer to ascertain nonverbal gestures and expressions that can be invaluable in qualitative research. Moreover, the follow-up probes did not specifically address the perceived quality of care postpartum women received from the healthcare providers. Omitting quality of care questions is a limitation because the literature documents that it is a factor that often influences how women characterize their care experiences with a healthcare provider or facility.

## 5. Conclusions

The COVID-19 pandemic exacerbated normal maternity-related stress. The COVID-19 mitigation strategies/behaviors that were mandated to curb the spread of COVID-19 posed challenges for postpartum women such as stress related to COVID-19, disruption of social life/support, disruption of expected healthcare services, uncertainty and fear about coronavirus, and poor maternal health outcome. Postpartum women reported increased anxiety, fear, frustration, lack of social support, long wait times to see the doctor, and shortened hospitalization after delivery. As we emerge from the COVID-19 pandemic or as COVID-19 prevention/mitigation strategies are lifted, there are many questions that continue to emerge related to maternal experiences, outcomes, and future directions for pregnancy care. In the aftermath of a global pandemic that challenged the public health workforce, healthcare systems, delivery of mental health services, and the freedoms and rights that we enjoy, there are many research questions and complex problems to address. The current study sought to better understand one aspect of maternal health in Mississippi. The lack of social support during pandemics like COVID-19 necessitates support groups in healthcare facilities to alleviate maternal healthcare difficulties.

## Figures and Tables

**Table 1.** Demographic Characteristics of Participants (*N* = 10).

Variables	Frequency
Age-group	
18–24 years old	3
25–34 years old	5
35–45 years old	2
Marital Status	
Married	6
Single	4
Education Level	
Associate degree	3
College degree	2
High school degree	4
Vocational training	1
Employment Status	
Unemployed (not looking for job)	1
Unemployed (looking for job)	3
Working full-time	4
Working part-time	1
Student	1
Household Income	
$10,000–$19,999	1
$20,000–$29,999	3
$30,000–$39,999	1
$40,000–$59,999	5
Insurance Status	
Medicaid	4
Other (marketplace)	2
Private	4
Delivery Status	
Late preterm	2
Past-due	2
Due date	6
Pre-existing Health Conditions	
ADHD	1
High blood pressure	1
Incompetence pelvic	1
None	7

Note. *N* = 10.

**Table 2.** Themes and Supporting Quotes.

Themes	Supporting Quotes
Stress related to COVID-19	“Somehow stressed, emotional stress of thinking of the uncertainty of what the virus would do to the child.” “It was tough, even though I enjoyed lockdown but just being confined to the house with a newborn Umm and still having to like work from home, it was tough. There was moment of frustrations.” “The COVID-19 restrictions when going to the hospital, it was a little bit stressful.” “I was kind of nervous about everything”, “it was a little bit stressful.” “It was a bit stressful you know with the sleepless nights and then trying to get better take care of the baby and do everything by yourself without people around, It was a little you know exhausting.” “I think it was a little more stressful because I had three kids to take care of, and the mask I was wearing also made it stressful. very uncomfortable and stressful because at every point in time, we also have to maskup, you’re sleeping and somebody comes into your room, you have to wake up and mask up.” “Umm stressful, anxious and frustrated and “I was moody, very moody but not a bad temper but kind of snappy.” “Frustrated and stressed and COVID was around and I had to deliver during COVID and I had to dislike wearing face mask.” “Wearing masks are stressful and uncomfortable and when you’re pregnant, especially when you start getting to the second and third trimester it’s very, when walking you know, sometimes you feel breathless.”
Disruption of social life/support	“They had to limit people coming into the room to just one person to know like I know before the pandemic, you will have your maybe your parents your spouse, but you just have to have only one person present. It was out, personally I wasn’t happy with it because you Know, I like that experience I wanted family and my spouse to be present but it just had to it was one or the other.” “It was bad to not have, like in during pre-COVID you could have as many people in the room as you want to, but during the COVID I am with one person and you know not having your mom or sister around when you deliver was kind of tough.” “I feel very much separated, being away from family and friends.” “I didn’t like the experience that much people where not allow to be with me during delivery, I actually wanted my step mom to be with me because this is my first child.” “I felt a little I guess alone because people didn’t visit that much and I couldn’t visit as well.” “I could only choose one person to be with me, but when I delivered him, it was his dad and my mom. It was just weird because I just remember everything, even my friends who have kids you know before COVID, everybody was just in the hospital, you know celebrating the baby and stuff, mine was just so different because you know you can’t invite people because of COVID.” “Umm not being able to go anywhere and not being able to have anyone come around, and whenever somebody kind of bring something they would leave it on the doorstep, so I feel like I have to play by yourself and like people had to avoid me at all costs, so I felt very isolated.” “It was a little sad, because due to social distancing I couldn’t go visit people and that made me sad.” “At home, I felt a little I guess alone because people didn’t visit that much.” “I feel very much separated, being away from family and friends.”
Disruption of expected healthcare services	“I like the way they handle their social distancing procedures but the waiting period was a long one.” “What I can say is that they won’t allow you to have your baby until like 39 weeks. They stopped inducing; before COVID-19 they can induce people Okay, but now because of COVID-19, they do not induce people; they want you to get ready, maybe baby almost you know. they will want you to come to the hospital for delivery when you are sure is your due day. This was because they don’t want too much people to be in the hospital at the same time.” “It was a long wait, the doctor I was seeing had a lot of patients, a patients was about to have a baby so he had to go there, so I either wait or reschedule.” “They made it in way that once you put to birth within 24 h you are to leave for another person to come in. It was a really tough one.” “They limit their appointment time or the number of people who are present during each appointment. Just to reduce like exposure to other people may have COVID.” “I guess unavailability of scheduling by not been able to get a visit within the week I needed to come because of the COVID it may have been delayed, so I will be coming two weeks after.” “So I was really concern I don’t want to get COVID while pregnant. My prenatal appointment was cancelled by the Doctor and rescheduled for another date.” “It was just really the delivery part and about like being sent home too early, because of COVID guidelines. I came to the hospital on 25th night, gave birth to my child on the 26th and I was back home on the 27th. I just feel like there wasn’t enough time to monitor and make sure I am OK before sending me home.” “I feel badly, because is a new protocols and people don’t stay in the hospital as long as they used to before COVID. Is like you had a baby, are you feeling good? Okay time for you to go home, I feel like I should have stayed longer, but I didn’t. I feel like I was failed honestly because first of all, they didn’t tell me that my blood pressure was high, or what signs to look out for if I need to come back or call, and you know something like that they didn’t educate me on it.” “I will give it a 7 over 10 because I think they could do better with follow up. It was just two postpartum follow-ups.”
Uncertainty and fear about coronavirus	“I was nervous. I think spend the first three to four months within, unless it was a doctor appointment, because I was afraid of the COVID.” “Umm it was scary, there was lot of uncertainties if I want to deliver in the hospital, so there was like my anxiety level high at sometimes.” “I am always scared of getting sick, so it won’t affect my baby, I try to stay six feet away from people.” “Not wanting to get COVID-19, there was like a barrier to not want to go out or not want to go see the doctor because other people may be sick.” “Hmm … It was it was scary because you know. It was something new and unfounded and I especially had concerns because, you know being pregnant, even though there was a vaccine, I chose not to take the vaccine, because I did, there was no research on you know the impact of it on pregnant women or the baby.” “There were some scary parts like getting COVID and all that, which made me to miss some of my doctor’s appointment you know.” “Somehow stressed, emotional stress of thinking of the uncertainty of what the virus would do to the child.”
COVID-19 mitigation strategies-associated poor maternal health outcome	“Um I think that’s where my postpartum depression came from, because you know, for a while you can’t leave the house and I actually have a baby, and it was just weird because it felt like this is how is going to be every single day and I barely go to anywhere anyway, because I was still terrified of COVID.” “I was breastfeeding also in pain after given birth I have forgotten what is called, it had to enter my body for 24 h, so I couldn’t get up for 24 h, it was miserable. I did experience postpartum depression.” “I had bad postpartum depression, so I was put on some different meds to help with that. I was on Zoloft for a little short period. I was just frustrated at that time because I had to take care of my newborn and my other two children myself.” “My care was a bit quick they somehow rushed me out of the hospital because of COVID. I was admitted again to the hospital, and I was told that I developed preeclampsia, so I had to stay in hospital for three days, because my blood pressure was extremely high.”

## Data Availability

The data presented in this study are available on request from the corresponding author.
